# Trends in the U.S. Childhood Emergency Department Visits for Fall-Related Fractures, 2001–2015

**DOI:** 10.7759/cureus.11629

**Published:** 2020-11-22

**Authors:** Carlos H Orces, Jacques Orces

**Affiliations:** 1 Rheumatology, Laredo Medical Center, Laredo, USA; 2 Emergency Department, Nicklaus Children's Hospital, Miami, USA

**Keywords:** children, falls, fractures, emergency department

## Abstract

Objective

The study's objective is to examine national trends in emergency department visits for unintentional fall-related fractures among children aged 0 to 19 years between 2001 and 2015.

Methods

The National Electronic Injury Surveillance System - All Injury Program was used to generate national estimates of fall-related fractures treated in emergency departments. Subsequently, according to demographic characteristics, body parts, and sport activities, age-adjusted fracture rates were calculated using the 2000 U.S. population as the standard. The joinpoint regression program was used to examine the average annual percent change in fracture rates during the study period.

Results

An estimated 7.9 million emergency department visits for fall-related fractures among U.S. children occurred between 2001 and 2015. Overall, upper extremity fractures accounted for 70% of the cases. Trend analyses demonstrated that fracture rates markedly decreased among children aged 10 to 15 years by -2.5% (95% C: -3.4% to -1.6%) per year. After adjusting for age, boys' fracture rates decreased annually by -1.9% (95% CI: -3.1% to -0.6%), whereas the average decrease in girls was less accentuated by -1.4% (95% CI: -1.8% to -1.0%) per year. Notably, forearm/wrist fracture rates decreased annually by -2.4% (95% CI: -2.9% to -1.9%) from 2004 onwards. In contrast, head and neck fracture rates significantly increased on average by 2.6% (95% CI: 1.3% to 3.9%) per year.

Conclusion

Childhood emergency department visits for fall-related fractures significantly decreased in the U.S. between 2001 and 2015. However, further research is needed to determine factors related to upward trends in head/neck fractures seen during the study period.

## Introduction

Falls are the leading cause of emergency department (ED) visits for childhood injuries in the United States (U.S.) [1]. Similarly, falls are the main mechanism of injury among children sustaining fractures, representing an estimated 14% of all U.S. pediatric injury-related ED visits in 2010 [2, 3]. Epidemiological studies have consistently demonstrated a greater incidence of fractures in boys than girls at all ages, with the peak of incidence at the age of 14 years in boys and 11 years in girls [3]. Overall, two-thirds of fractures among children occur in the upper extremities, and forearm fracture is the most frequently reported fracture in both genders [4]. In addition, childhood fractures are associated with days of leisure-time activity restriction, varying according to the type of fracture [5].

Although research data in pediatric fracture trends are scarce, a few studies mostly conducted in Scandinavian countries have reported contradictory results over time. For instance, in Helsinki, Finland, the incidence of fractures in children aged 0 to 15 years significantly decreased between 1983 and 2005 [6]. In contrast, fracture rates among children increased by 13% during the period 1998-2007 in Northern Sweden, whereas decreased rates predominantly among girls were reported between 1993-1994 and 2005-2006 in Malmö, Sweden [2, 7]. 

Notably, a recent retrospective analysis of ED attendance for children and adolescents fall-related injuries in Hong Kong described that fall-related fracture rates significantly increased between 2001 and 2012 [8]. The increase in the incidence of fractures in that latter study was particularly attributed to upward trends in wrist/hand and ankle/foot fracture rates. Whether similar childhood fall-related fracture rates have occurred in the U.S. is currently unknown. Thus, the present study aimed to examine national trends in ED visits for fall-related fractures in children and adolescents between 2001 and 2015.

## Materials and methods

The National Electronic Injury Surveillance System - All Injury Program (NEISS-AIP) provides national incidence estimates of all types and external causes of nonfatal injuries and poisonings treated in U.S. hospital emergency departments (EDs). Since July 2000, data on injury-related visits are being obtained from a national sample of 66 of 100 NEISS hospitals, which were selected as a stratified probability sample of hospitals in the United States and its territories with a minimum of six beds and 24-hour EDs. The NEISS-AIP collects data on age, race, Hispanic origin, gender, principal injury diagnosis, primary body-part affected, the place where the injury occurred, disposition at discharge from the EDs, and mechanism of injury. The present analysis was limited to EDs visits for unintentional fall-related fractures among children aged 0 to 19 years between 2001 and 2015. National estimates are considered unstable and potentially unreliable if the number of records is based on fewer than 20 NEISS-AIP cases or national estimates fewer than 1200 (based on weighted data). The NEISS-AIP public use files were downloaded from the Inter-University Consortium for Political and Social Research [9].

Statistical analysis

The NEISS-AIP sampling weights were used to obtain unbiased, nationally representative estimates of fall-related fractures among children aged 0 to 19 years. The sample weight has been adjusted for hospital nonresponse within each NEISS-AIP sample stratum, and changes in the number of EDs visits annually in the sampling frame of U.S. hospital EDs. July 1^st^ population estimates were used as the denominator to calculate age-specific fracture rates (0-4, 5-9, 10-14, 15-19 years) per 100,000 children. Age-specific fracture rates were then directly standardized to the 2000 U.S. population [10]. Trends in fall-related fracture rates were examined by age-group, sex, primary body part-grouped (head/neck, upper trunk, arm/hand, lower trunk, and leg/foot), upper extremity (hand/finger, forearm/wrist, and elbow/arm/shoulder), and sports-related activities. The joinpoint regression program software version 4.7.0.0 (Surveillance Research Program, National Cancer Institute, US) was used to identify points where a statistically significant change occurred in the linear slope of fracture rates. The results are presented as the average annual percent change (AAPC) with their corresponding 95% confidence interval (95% CI). The AAPC in rates is a summary measure of the trend over a pre-specified fixed interval. This measure describes the average annual percent changes over a period of multiple years, which is valid even if the joinpoint model indicates that there were changes in trends during those years, which are reported as the annual percent changes (APC) in rates of the period [11]. A maximum of three joinpoints were allowed for each estimate. All statistical analyses were performed using SPSS Complex Sample software, version 17 (SPSS Inc, Chicago, Illinois, USA), which incorporated sampling weights to obtain nationally representative estimates of EDs visits for fall-related fractures among children.

## Results

Based on 180,093 cases, an estimated 7.9 million ED visits for unintentional fall-related fractures in children and adolescents occurred between 2001 and 2015. Overall, the estimated number of ED visits for fall-related fractures decreased by 20%, from 554,000 in 2001 to 443,00 in 2015. Table 1 shows the characteristics of children treated in EDs during the study period. In general, boys, children aged 5-14 years, and non-Hispanics sustained more frequently fall-related fractures. Notably, arm/hand fractures accounted for 70% of the ED visits for all fractures. By place of injury, fractures occurred similarly at home or school. Of relevance, only 5% of children were hospitalized for these injuries.

**Table 1:** Fall-related fractures among children treated in emergency departments, 2001-2015 AMA - against medical advice Parenthesis represents standard errors (SE)

Characteristics	No. of cases	% (SE)
Age groups (years)		
≤ 4	44,200	20.7 (0.7)
5 - 9	60,442	32.3 (0.5)
10 - 14	55,014	32.5 (0.6)
15 - 19	20,437	14.5 (0.5)
Gender		
Boys	107,393	59.8 (0.5)
Girls	72,700	40.2 (0.5)
Hispanic origin		
Hispanic	20,722	8.7 (2.2)
Non-Hispanic	159,371	91.3 (2.2)
Primary body part-grouped		
Head/neck	6,250	3.1 (0.2)
Upper trunk	15,052	9.1 (0.2)
Arm/hand	125,840	70.1 (0.5)
Lower trunk	1,328	0.9 (0.0)
Leg/foot	31,623	16.8 (0.4)
Place of injury		
Unknown	49,077	23.7 (3.3)
Home	61,578	35.4 (2.1)
School	57,959	33.8 (1.5)
Street	4,543	3.2 (0.8)
Other property	6,788	3.8 (0.3)
Farm	148	0.1 (0.0)
Sports-related injury		
Yes	80,504	46.3 (1.2)
No	99,589	53.7 (1.2)
Disposition		
Treated/released	163,383	91.7 (0.6)
Transferred/released	2,232	2.6 (0.3)
Hospitalized	13,622	5.3 (0.7)
Observation	643	0.3 (0.1)
AMA	142	0.1 (0.0)
Unknown	61	0.0 (0.0)
Seasons		
Winter	34,159	20.2 (0.8)
Spring	50,027	27.3 (0.4)
Summer	49,845	26.5 (0.5)
Fall	46,062	26.0 (0.4)

**Figure 1:**
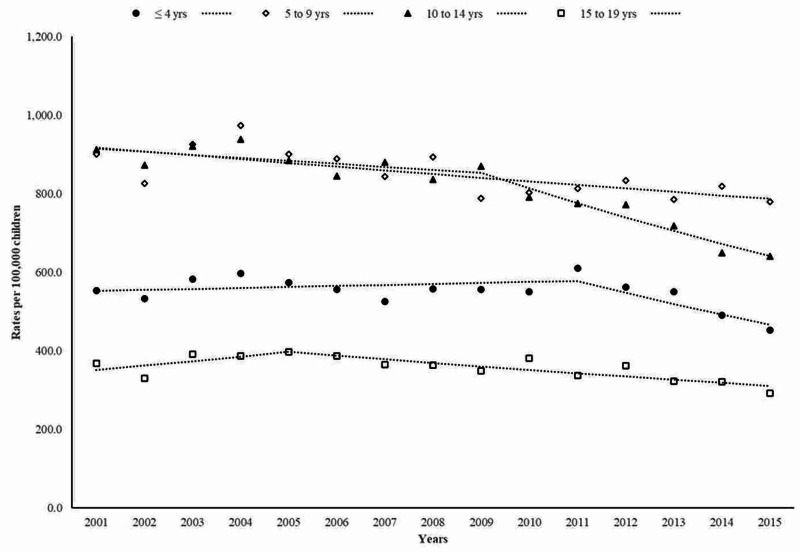
Trends in emergency department visits for fall-related fractures

As shown in Figure 1, ED visits for fall-related fracture rates significantly decreased among children aged 10 to 14 years on average by -2.5% (95% CI: -3.4% to -1.6%) per year. Notably, a marked downward trend in fracture rates by -4.7% (95% CI: -6.3% to -3.0%) per year was seen in this age-group between 2009 and 2015. Fracture rates among children ≤ four years and aged from five to nine years similarly decreased at an AAPC of -1.2% (95% CI: -2.3% to -0.1%) and -1.1% (95% CI: -1.7% to -0.5%) during the study period, respectively. 

**Figure 2:**
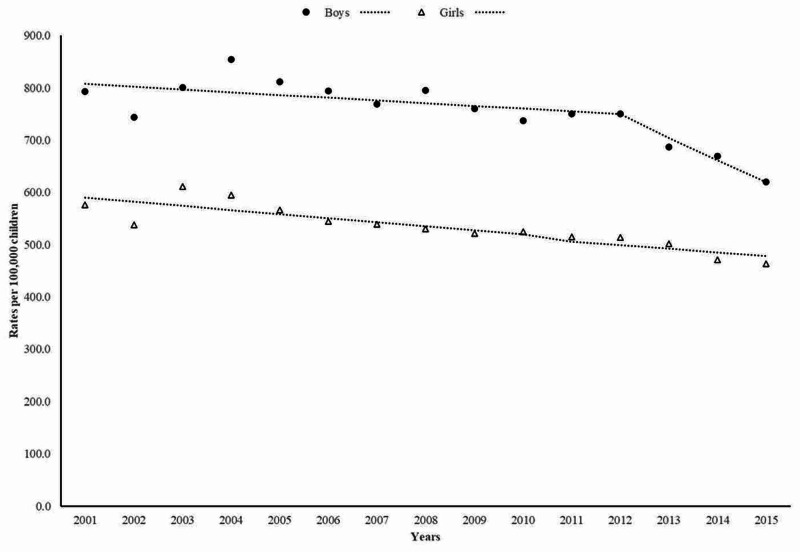
Trends in emergency department visits for fall-related fractures in boys and girls

As shown in Figure 2, age-adjusted fall-related fracture rates in boys decreased annually by -1.9% (95% CI, -3.1% to -0.6%), whereas the decrease in rates among girls was less accentuated, corresponding to - 1.4% (95% CI, -1.8% to -1.0%) per year. 

Table 2 describes trends in age-adjusted fall-related fracture rates according to primary body parts. Overall, arm/hand fracture rates decreased by -1.6% (95% CI: -2.5% to -0.7%) per year. Notably, a marked downward trend in these fractures by -4.1% (95% CI: -7.1% to -1.0%) per year occurred from 2012 onwards. Similarly, leg/foot fracture rates annually decreased by -1.4% (95% CI: -3.0 to 0.3). In contrast, head/neck fracture rates significantly increased by 2.6% (95% CI: 1.3% to 3.9%) per year. 

**Table 2:** Trends in childhood emergency department visits for fall-related fractures according to primary body parts AAPC - average annual percent change in rates; APC - annual percent change in rates * Significantly different from zero at alpha = 0.05 level

Body parts	AAPC (95% CI)	APC (95% CI)	Trend 1	APC (95% CI)	Trend 2
Head/neck	2.6% (1.3% to 3.9%)*				
Upper trunk	-0.9% (-1.8% to -0.1%)*				
Arm/hand	-1.6% (-2.5% to -0.7%)*	-1.7% (-2.5% to -0.9%)*	2004-2012	-4.1% (-7.1% to -1.0%)*	2012-2015
Lower trunk	0.1% (-1.2% to 1.4%)				
Leg/foot	-1.4% (-3.0% to 0.3%)	-3.2% (-4.5% to 1.8%)*	2005-2015		

**Figure 3:**
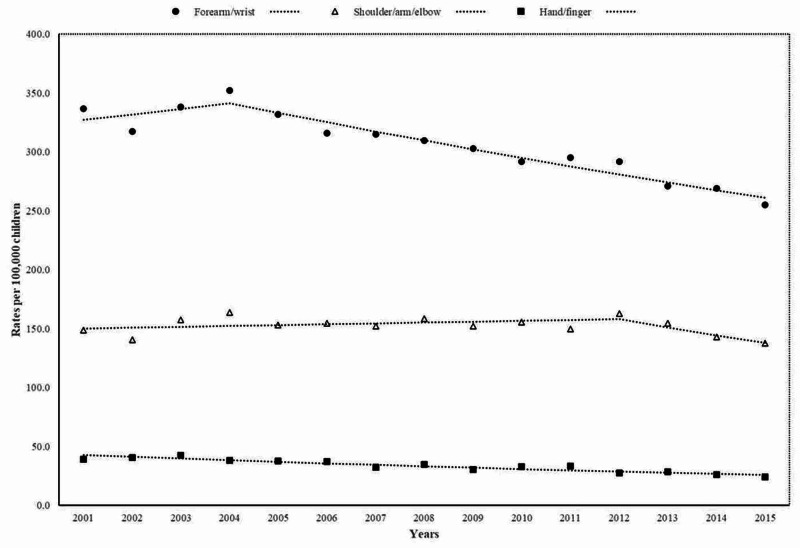
Trends in emergency department visits for fall-related upper extremity fractures

Figure 3 shows trends in upper extremity fractures between 2001 and 2015. Notably, forearm and/or wrist fracture rates decreased on average by -1.6% (95% CI: -2.4% to -0.8%) per year. Moreover, these fractures markedly decreased annually by -2.4% (95% CI: -2.9% to -1.9%) from 2004 onwards. Likewise, hand/finger fracture rates linearly decreased on average by -3.5% (95% CI: -4.3% to -2.8%) per year. In contrast, shoulder, arm, and/or elbow fracture rates remained unchanged during the same period. 

**Figure 4:**
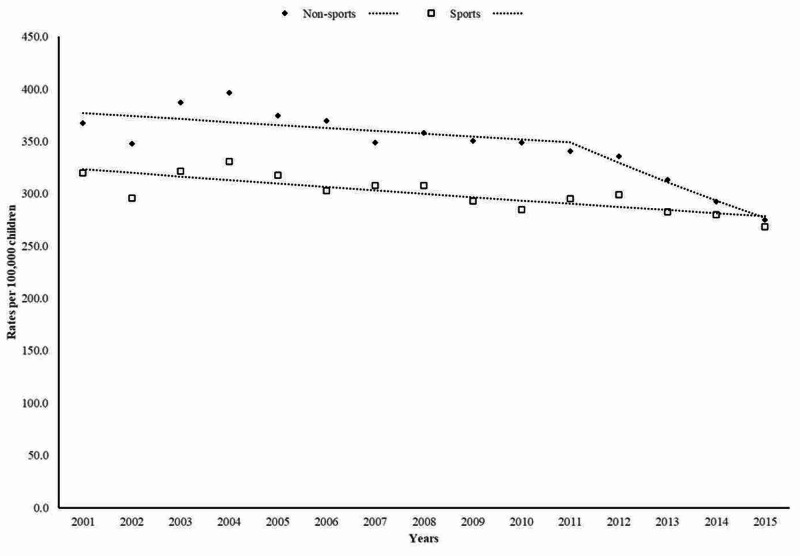
Trends in emergency department visits for fall-related fractures according to sports-related activities

As shown in Figure 4, non-sports fracture rates decreased on average by -2.2% (95% CI: -3.3% to -1.1%) per year. In addition, a marked decrease in annual rates by -5.6% (95% CI: -9.2% to -1.9%) was seen between 2011 and 2015. Similarly, fall-related fractures associated with sports activities decreased at a slower rate by -1.1% (95% CI: -1.5% to -0.6%) per year.

## Discussion

In a nationally representative sample of ED visits for injuries, childhood fall-related fractures significantly decreased between 2001 and 2015. The decrease in the incidence of ED visits for fall-related fractures in children and adolescents was attributed to downward trends in fractures seen in the age group of 10 to 14 years, among boys, upper limb fractures, and particularly forearm and/or wrist fractures. 

The present findings are consistent with those reported by Mäyränpää et al. in a study conducted among children aged 0 to 15 years, in which fracture rates decreased by 18.5% in Helsinki, Finland between 1983 and 2005. Moreover, the greatest decrease in fracture incidence in that study was seen among children aged 10 to 13 years [6]. Conversely, a recent population-based trend analysis of fall-related ED visits for fractures among children aged 0 to 19 years in Hong Kong reported that adjusted fracture rates increased at an annual percentage of 1.3% between 2001 and 2012, which contrast with the present study results. Although the reasons for the diverging trends found in this latter and our study remain to be elucidated, the overall upward trend in childhood ED visits for fall-related fractures in Hong Kong over the past decade may have been attributed to increased incidence of ankle/foot and wrist/hand fractures [8]. 

Notably, ED visits for fall-related forearm/wrist fractures in children significantly decreased during the study period. Moreover, a marked annual decrease in fracture rates by 2.4 % was seen from 2004 onwards, which contrasts with those reported in Scandinavian countries, Hong Kong, and Olmsted County, Minnesota [6, 8, 12, 13]. Indeed, a registry study from southern Sweden demonstrated that the age-standardized distal forearm fracture rate increased by 2.2% per year between 1999 and 2010. In addition, that latter study demonstrated a 50% higher incidence rate of distal forearm fractures compared with those reported during the period 1950-1965 [12]. Of relevance, the only population-based study conducted to examine the incidence of childhood EDs visits for fall-related fractures described that forearm/elbow and wrist/hand fractures increased in Hong Kong by 1.4% and 3% annually between 2001 and 2012, respectively [8]. 

Previous studies have described that between 36% and 50% of childhood fractures are related to sports activities suggesting that the higher bone mass associated with increased physical activity in children does not compensate for the increased exposure to injuries [14, 15]. The present findings indicate that about 46% of ED visits for fall-related fractures among children are related to sports activities, which have linearly decreased over time. Shah et al., in a retrospective study using data from the U.S. Consumer Product Safety Commission's (CPSC) NEISS, also reported a decline in the number of ED visits for bicycle- and basketball-related wrist fractures, whereas soccer-related wrist fractures increased between 1998 and 2013 [16]. In contrast, Bayt et al., using the same database, reported that ED visits for selected sports-related injuries significantly increased among children aged 5-18 years between 2001 and 2013. Overall, football, basketball, soccer, and baseball resulted in 74.7% of the total national estimate for sports-related injuries [17]. Discrepancies between our results and those reported using the CPSC-NEISS data may be related to the consumer product-specific codes at the time of injury and no information regarding the mechanism of injury [16]. 

Notably, fall-related head/neck fractures among children significantly increased on average by 2.6% annually between 2001 and 2015. Consistent with our findings, a recent analysis of the NEISS-AIP data also reported that ED visits for sports or recreational-related traumatic brain injury significantly increased between 2001 and 2012. Overall, 70% of traumatic brain injury (TBI) occurred among persons 19 years or younger. Moreover, the largest number of TBI among males occurred during bicycling, football, and basketball, while in females during bicycling, playground activities, and horseback riding. Although the precise reason for upward trends in childhood fall-related head/neck fractures is unknown, it has been suggested that may be associated with increased awareness of TBI through increased media exposure and availability of CT or MRI scanners [18]. 

A potential explanation for the downward trends in childhood ED visits for fall-related fractures seen in the present study may be related to increased physical activity among children over time. Among U.S. youth, a recent analysis of secular trends in physical activity reported that the percentage of high school attending daily physical education classes declined from 42% in 1991 to 31% in 2011. However, high school sports team participation in boys remained unchanged at about 50% from 1970 to 2012, whereas sports participation in girls has increased steadily since 1971 [19]. The relevance of school-based physical activity on childhood fracture was recently demonstrated among participants in the Pediatric Osteoporosis Prevention study, a Swedish prospective controlled intervention study in which children aged from six to eight years who participated in daily moderate physical activity intervention had a 52% lower fracture risk at eight years of follow up compared with their counterparts who had 60 minutes of physical activity per week. The authors concluded that long-term physical activity might positively affect bone mass, muscle strength, and neuromuscular function, leading to a reduction in fracture risk [20, 21]. In contrast, a dose-dependent time spent on television, computer, and video viewing in children was positively associated with wrist and forearm fractures [22]. 

The present study has several limitations. First, fall-related fractures may have been underestimated since the NEISS-AIP does not include children treated in other outpatient settings. Similarly, fractures may be underestimated because only the principal diagnosis and the primary body part injured are collected in NEISS-AIP. Therefore, more serious injuries may have been recorded first. Second, the NEISS-AIP coding system has a ﬁxed number of categories for the primary body part affected, and the International Classification of Diseases, Clinical Modification (ICD-CM) diagnosis codes are not available in the medical record. Consequently, specific types of fractures may not be identified. Third, NEISS-AIP data do not include geographic data. Finally, there is no information on known risk factors for fractures, such as bone mineral density, calcium, vitamin D intake, or physical activity, contributing to the downward trend in ED visits for childhood fractures. 

## Conclusions

Emergency department visits for unintentional fall-related fractures among children and adolescents in the U.S. significantly decreased between 2001 and 2015. However, further research is needed to determine factors related to increasing childhood head/neck fractures seen during the same period.

## References

[1] (2020). WISQARS™ Leading causes of nonfatal injury reports, 2000 - 2018. of nonfatal injury reports.

[2] Hedström EM, Svensson O, Bergström U, Michno P (2010). Epidemiology of fractures in children and adolescents. Acta Orthop.

[3] Naranje SM, Erali RA, Warner WC Jr, Jeffrey SR, Derek KM (2016). Epidemiology of pediatric fractures presenting to emergency departments in the United States. J Pediatr Orthop.

[4] Cooper C, Dennison EM, Leufkens HG (2004). Epidemiology of childhood fractures in Britain: a study using the general practice research database. J Bone Miner Res.

[5] Kopjar B, Wickizer TM (1998). Fractures among children: incidence and impact on daily activities. Inj Prev.

[6] Mäyränpää MK, Mäkitie O, Kallio PE (2010). Decreasing incidence and changing pattern of childhood fractures: a population-based study. J Bone Miner Res.

[7] Tiderius CJ, Landin L, Düppe H (1999). Decreasing incidence of fractures in children: an epidemiological analysis of 1,673 fractures in Malmö, Sweden, 1993-1994. Acta Orthop Scand.

[8] Lee JC, Tung KT, Li TM (2017). Fall-related attendance and associated hospitalization of children and adolescents in Hong Kong: a 12-year retrospective study. BMJ Open.

[9] (2020). ICPSR find & analyze data. https://www.icpsr.umich.edu/icpsrweb/ICPSR/search/studies.

[10] (2020). CDC Wonder. https://wonder.cdc.gov/.

[11] (2020). Joinpoint. https://surveillance.cancer.gov/help/joinpoint.

[12] Jerrhag D, Englund M, Petersson I (2016). Increasing wrist fracture rates in children may have major implications for future adult fracture burden. Acta Orthop.

[13] Khosla S, Melton LJ 3rd, Dekutoski MB (2003). Incidence of childhood distal forearm fractures over 30 years: a population-based study. JAMA.

[14] Lyons RA, Delahunty AM, Kraus D (1999). Children's fractures: a population-based study. Inj Prev.

[15] Clark EM, Ness AR, Tobias JH (2008). Vigorous physical activity increases fracture risk in children irrespective of bone mass: a prospective study of the independent risk factors for fractures in healthy children. J Bone Miner Res.

[16] Shah NS, Buzas D, Zinberg EM (2015). Epidemiologic dynamics contributing to pediatric wrist fractures in the United States. Hand.

[17] Bayt DR, Bell TM (2016). Trends in paediatric sports-related injuries presenting to US emergency departments, 2001-2013. Inj Prev.

[18] Coronado VG, Haileyesus T, Cheng TA (2015). Trends in sports- and recreation-related traumatic brain injuries treated in US emergency departments: The National Electronic Injury Surveillance System-All Injury Program (NEISS-AIP) 2001-2012. J Head Trauma Rehabil.

[19] Bassett DR, John D, Conger SA (2015). Trends in physical activity and sedentary behaviors of United States youth. J Phys Act Health.

[20] Cöster ME, Fritz J, Nilsson JÅ (2017). How does a physical activity programme in elementary school affect fracture risk? A prospective controlled intervention study in Malmo, Sweden. BMJ Open.

[21] Karlsson MK, Rosengren BE (2020). Exercise and peak bone mass. Curr Osteoporos Rep.

[22] Ma D, Jones G (2003). Television, computer, and video viewing; physical activity; and upper limb fracture risk in children: a population-based case control study. J Bone Miner Res.

